# Potential impact of GLP-1 receptor agonists on male fertility: a fable of caution

**DOI:** 10.3389/fphys.2024.1496416

**Published:** 2024-11-18

**Authors:** Stefan S. Du Plessis, Temidayo S. Omolaoye, Walter D. Cardona Maya

**Affiliations:** ^1^ College of Medicine, Mohammed Bin Rashid University of Medicine and Health Sciences, Dubai Health, Dubai, United Arab Emirates; ^2^ Reproduction Group, Department of Microbiology and Parasitology, Faculty of Medicine, Universidad de Antioquia, Medellín, Colombia

**Keywords:** GLP-1 - glucagon-like peptide-1, GLP-1 receptor agonists, male fertility, sperm parameters, sexual health

The recent surge in the use of glucagon-like peptide-1 (GLP-1) receptor agonists, such as Mounjaro (tirzepatide) licensed by Eli Lilly and Company, and Ozempic (semaglutide) licensed by Novo Nordisk A/S., for the management of type 2 diabetes and obesity, has sparked significant interest in their broader physiological effects (Jensterle et al., 2019; Syed, 2022; Watanabe et al., 2024). While these medications are celebrated for their role in improving glycemic control and aiding weight loss, their potential impact on male fertility remains an underexplored area that warrants attention.

GLP-1 receptor agonists work by mimicking the action of the GLP-1 hormone, which plays a crucial role in regulating blood sugar levels and appetite. The mechanisms through which these drugs exert their effects, including enhancing insulin secretion, slowing gastric emptying, and promoting satiety, have been well documented (Shaefer et al., 2015; Drucker, 2018). However, emerging evidence suggests that GLP-1 receptors are also present in male reproductive tissues, such as the testes, raising questions about the potential implications of these drugs on male fertility (Jeibmann et al., 2005; Alves et al., 2016; Rago et al., 2020).

The interplay between metabolic health and reproductive function is well established, with conditions like obesity and diabetes often linked to reduced sperm quality and male infertility (Cabler et al., 2010; Du Plessis et al., 2010; Service et al., 2023). By improving metabolic parameters, GLP-1 receptor agonists could theoretically contribute to better reproductive outcomes. However, this is not entirely the case, especially from previously investigated GLP-1 receptor agonists, such as liraglutide, where controversy exist in the findings. Also, the direct effects of these medications on sperm function, hormone levels, and overall male fertility remain largely unexplored in clinical studies.

A search conducted on August 27, 2024, in the PubMed database using the MeSH terms: “Glucagon-Like Peptide-1” OR “Glucagon-Like Peptide-1 Receptor” AND Fertility identified 50 published articles between 2011 and 2024. Upon reviewing the titles and abstracts, only 16 articles discussed or evaluated the potential effects on the male factor, either in humans or other models.

High-fat diet-fed mice exhibited lower serum testosterone, and administration of GLP-1 receptor agonists did not restore these levels. However, sperm motility, mitochondrial activity, and sperm DNA fragmentation improved (Zhang et al., 2015). Other animal studies have provided insights indicating that GLP-1 receptor activation may influence testicular function and spermatogenesis (Jensterle et al., 2019). In humans, patients treated with liraglutide exhibited a significant increase in serum testosterone and significant improvement in conventional sperm parameters such as sperm count, progressive motility and normal morphology (La Vignera et al., 2023). Additionally, findings from a randomized, double-blind, controlled trial showed that after an initial 8-week diet weight loss, the percentage of men with oligozoospermia reduced, and was sustained over a year in participants who maintained their weight loss (Andersen et al., 2022). Another study which evaluated healthy men of normal weight reported no negative effects of a 4-week treatment with dulaglutide on sexual desire, hormone levels, or sperm parameters (Lengsfeld et al., 2024).

Conversely, a study reported the adverse effect of liraglutide on sperm quality, which was restored after 5 months of discontinuation of liraglutide. However, it did not reach the initial values for semen volume, or sperm concentration and motility (Fontoura et al., 2014). Another study showed that GLP-1 signalling downregulated testosterone production and potentially impacts sperm quality (Jeibmann et al., 2005).

Given the importance of fertility preservation in patients of reproductive age, it is crucial to investigate the long-term effects of GLP-1 receptor agonists on male reproductive health. Additionally, gametogenesis is a long process, taking approximately 3 months, coupled with its high sensitivity to biological variations, such that minor changes could have significant impact on the population. This further iterate the need for dedicated clinical studies that examine parameters such as sperm count, motility, morphology, and hormonal profiles in men undergoing treatment with these medications. Additionally, it is essential to consider the potential for differential effects based on dosage, treatment duration, and underlying health conditions.

In conclusion, while GLP-1 receptor agonists offer significant benefits in managing metabolic disorders, their potential impact on male fertility remains an area of concern that deserves further research. Understanding these effects is vital not only for the informed use of these medications but also for the broader implications of reproductive health in men undergoing treatment for chronic conditions. The temporary benefits of any treatment should not obscure the understanding of potential long-term harm that could impact fertility; in some cases, the treatment may be more detrimental than the condition itself. We advocate for increased attention to this topic in future clinical trials and encourage the scientific community to explore this promising yet uncharted territory in reproductive medicine.
